# Comparison of the Effects of LSP Treatment on Wrought and Additive Manufactured Ti-6Al-4V Samples

**DOI:** 10.3390/ma19081582

**Published:** 2026-04-15

**Authors:** Irvin Alejandro Guillen-Virgen, Gilberto Gomez-Rosas, Eduardo Castañeda-Paredes, Martha Guadalupe Arredondo Bravo, Olga Klimova-Korsmik, Marina Gushchina

**Affiliations:** 1 University Centre for Exact Sciences and Engineering (CUCEI), University of Guadalajara, Blvd. Marcelino García Barragan #1421, Guadalajara 44430, Mexico; irvin.guillen8883@alumnos.udg.mx; 2 Department of Physics, University Centre for Exact Sciences and Engineering (CUCEI), University of Guadalajara, Blvd. Marcelino García Barragan #1421, Guadalajara 44430, Mexico; martha.arredondo@academicos.udg.mx; 3 Department of Electrical Mechanical Engineering, University Centre for Exact Sciences and Engineering (CUCEI), University of Guadalajara, Blvd. Marcelino García Barragán #1421, Guadalajara 44430, Mexico; eduardo.castaneda@academicos.udg.mx; 4 Institute of Laser and Welding Technologies, Saint-Petersburg State Marine Technical University, Saint-Petersburg 190121, Russia; o.klimova@ltc.ru (O.K.-K.); gushinam@yandex.ru (M.G.)

**Keywords:** laser shock peening, Ti-6Al-4V, residual stress, microhardness, microstructure, additive manufacturing, wrought manufacturing, mechanical properties

## Abstract

Laser shock peening (LSP) is a surface treatment technique focused on improving the mechanical performance of metal components by inducing compressive residual stresses. This research evaluated the effects of LSP on a Ti-6Al-4V alloy, an α + β titanium alloy manufactured by wrought and additive manufacturing used in biomedical and aerospace applications. Samples manufactured by conventional processes and additive manufacturing were treated under the following conditions: Pulse width of 6 ns, wavelength of 1064 nm, scan density of 2500 pulses/cm^2^, pulse energy of 0.750 J, and repetition frequency of 10 Hz. The mechanical response was evaluated in terms of residual stress, microhardness, and microstructure before and after treatment. The results showed significant improvements, reaching compressive residual stress fields of up to −800 MPa and a 22% increase in microhardness, and grain refinement from 18.16 μm to 5.54 μm. These results confirm the effectiveness of LSP in improving the surface integrity and mechanical behavior of Ti64 components, regardless of their manufacturing method.

## 1. Introduction

Laser shock peening (LSP) treatment is a technique developed for treating metal surfaces. When applied correctly to a material, LSP improves the mechanical properties of the treated component due to the generation of compressive residual stresses in the metal matrix of the material [1,2,3,4,5]. After the LSP treatment was developed, the main research focus was on understanding the mechanisms for its application to different metallic materials such as cast iron, aluminum alloys, titanium and its various alloys, nickel-based superalloys, etc., because one of the main objectives pursued in the industry is to obtain metal parts with a long service life in the face of various types of mechanical failures (fatigue, corrosion, and wear) [6,7,8]. The main objective of LSP treatment is to generate compressive residual stress at a certain depth below the surface of the treated material, thereby improving its mechanical properties such as hardness and fatigue resistance. This objective can be achieved by generating plasma on the surface after the impact of a high-energy laser pulse lasting a few nanoseconds [9,10]. Compared to conventional surface treatments (shot blasting), LSP can not only generate higher compressive residual stresses (of hundreds of MPa) and greater depth (close to 1 mm), but can also refine the grain in the first layers of the material undergoing treatment [11,12]. If the LSP treatment is generated in a vacuum, the plasma generated expands freely. This procedure is known as direct ablation. Conversely, if a layer of material that is transparent to laser radiation (confining layer) is added to the treatment, the plasma can be confined and its expansion slowed. This procedure is known as confined ablation [13,14].

Recent advancements in laser shock peening (LSP) have increasingly recognized this technique as a critical stage within hybrid manufacturing frameworks to ensure surface integrity. In this context, the precise control of laser parameters is essential to mitigate surface defects and optimize roughness, particularly when addressing the inherent limitations of traditional manufacturing [15]. Furthermore, the stability of the induced residual stress fields remains the cornerstone of high-performance applications, as modern experimental validations emphasize the superior depth and thermal stability of LSP-induced compression layers compared to other mechanical treatments [16]. However, the comparative effect of these mechanisms on samples fabricated through additive manufacturing versus conventional methods requires further investigation.

Previous studies have demonstrated the benefits of LSP treatment in various titanium alloys. Some examples of this are Cesar A. Reynoso-Garcia et al. [17], who demonstrated that Ti-6Al-4V alloy subjected to LSP treatment with a wavelength of 1064 nm improves its mechanical properties due to a 257.14% increase in residual compressive stresses compared to an untreated sample. These benefits are notably relevant for complex geometries, where the use of femtosecond pulses (FLSP) has proven effective in Ti-6Al-4V thin-walled structures, achieving a 27.1% increase in surface hardness and a 12.2% improvement in fatigue limit through controlled grain refinement and subgrain formation [18]. Xiang fan Nie et al. [11] characterized the effects of LSP surface treatment on the titanium alloy known as TC6, demonstrating an increase in the Vickers hardness of this alloy. For their part, K. Dyer et al. [19] in 2024 studied the effect of LSP on surface finish and its improvement in the fatigue of Ti-6Al-4V parts manufactured by additive manufacturing, demonstrating that the use of a mechanical profilometer did not detect changes in the samples, but destructive analysis showed notable reductions in roughness after LSP, indicating that contact instruments do not capture the depth valleys typical of additive manufacturing well. It also demonstrated an increase in fatigue life in parts with greater surface porosity.

In the research conducted by E. Maleki et al. [8], LSP was carried out on AlSi10Mg manufactured by L-PBF. They found that it reduces roughness, hardens the surface, generates compressive stress and achieves a significant improvement in corrosion resistance (reduction in corrosion rate of around 50%). For their part, Wei Guo et al. [20] studied the effect of LSP treatment on the surface microstructure of Ti-6Al-4V alloy manufactured using the LAM (Laser Additive Manufacturing) technique, demonstrating grain refinement in the α phase from 33.6 to 24.3 μm, which we attribute to the generation of compressive residual stresses that reduce the spacing between crystal lattices, which in turn has a direct impact on improving the hardness of the material. Before undergoing treatment, the hardness was 361.0 HV, and after irradiation, it increased to 419.6 HV, an increase of 16.5%. It is fundamental to distinguish that, unlike the equilibrium microstructure found in WR Ti-6Al-4V, additive manufactured components exhibit a distinct non-equilibrium microstructural evolution. As demonstrated by Yang et al. [21], the rapid solidification rates inherent to the manufacturing process trigger the formation of an acicular α′ martensite phase, which significantly alters the material’s initial state and its subsequent response to surface treatments like LSP. However, comparative studies between LSP-treated wrought and LB-DED Ti-6Al-4V under identical processing conditions remain limited. Therefore, this work aims to systematically compare microstructural evolution, residual stress distribution, and hardness response in both manufacturing routes.

The main objective of this research is to compare the effect of LSP treatment on the microstructure and mechanical properties of Ti-6Al-4V alloy parts manufactured by wrought and additive manufacturing processes, using X-ray fluorescence (XRF) spectroscopy, X-ray diffraction (XRD), residual compressive stresses (hole drilling) and hardness (HV). The LSP treatment used scan densities of 2500 pulses/cm^2^, wavelength of 1064 nm, pulse width of 6 ns, energy per pulse of 0.750 J and a repetition rate of 10 Hz.

## 2. Materials and Methods

### 2.1. Preparation of Samples Before Treatment

The Ti-6Al-4V alloy, also known as Ti64, is an α + β alloy composed of aluminum and vanadium (shown in Table 1); the mechanical properties are shown in Table 2 [22,23,24,25]. Additive manufacturing is defined in the joint ISO/ASTM standards as “the process of joining materials to build objects from 3D model data, typically layer by layer,” as opposed to subtractive and formative manufacturing methodologies. The ISO/ASTM standards classify additive manufacturing processes into seven categories [26,27].

One of the methods used for the manufacture of metal parts using additive manufacturing is the laser-based directed energy deposition (LB-DED) technique. This uses lasers as a source of thermal energy to melt the metal material, demonstrating its ability to manufacture and repair complex components [21].

However, it has been reported that this type of manufacturing process generates residual stress strains that tend to deteriorate the mechanical properties of components manufactured using this method and, therefore, their possible future applications [28,29].

Two types of samples were used for the LSP treatment application. The first was a sample obtained from a Ti-6Al-4V alloy plate with the following dimensions: 50 mm × 50 mm × 9 mm. According to the supplier’s technical specifications, this material was supplied in the mill-annealed condition (processed at 704–790 °C for 1–4 h, followed by air cooling) [17]. This test piece was identified as WR Ti-6Al-4V (Wrought Ti-6Al-4V). The chemical and mechanical properties are shown in Table 1 and Table 2, respectively.

**Figure 1:**
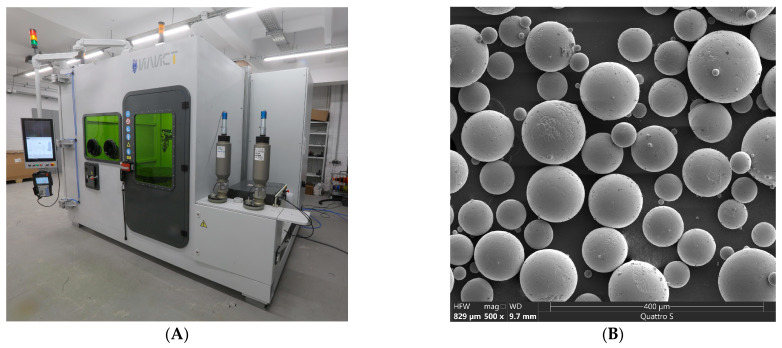
(**A**) Robotic complex ILIST-L for DED; (**B**) SEM images of Ti-6Al-4V powder surface for DED process.

The second sample was obtained using the additive manufacturing method and was designed with the following dimensions: 50 mm × 50 mm × 9 mm. It was produced using the LB-DED technique (Institute of Laser and Welding Technologies, Saint Petersburg, Russia), employing the ILIST-L direct metal deposition (DMD) machine (Figure 1A) manufactured by the Institute of Laser Technologies and Welding at Saint Petersburg State Marine Technical University (ILWT, SMTU, Saint Petersburg, Russia) under the following DMD process parameters: a power of 1900 W and a processing tool speed of 20 mm/s, a *Z*-axis displacement of 0.8 mm and a width displacement of 2.5 mm. Ti-6Al-4V alloy powder with a fraction of 45–140 μm, manufactured using PREP technology (Figure 1B), was used as the base material. The chemical composition of the Ti-6Al-4V alloy powder is shown in Table 3. No discrepancies were detected according to ASTM F3001-14, identified as AM Ti-6Al-4V [30]. Both samples were machined and ground with 120 grit/cm^2^ SiC abrasive paper to reduce the residual stresses resulting from the manufacturing process.

### 2.2. Experimental Setups

**Figure 2:**
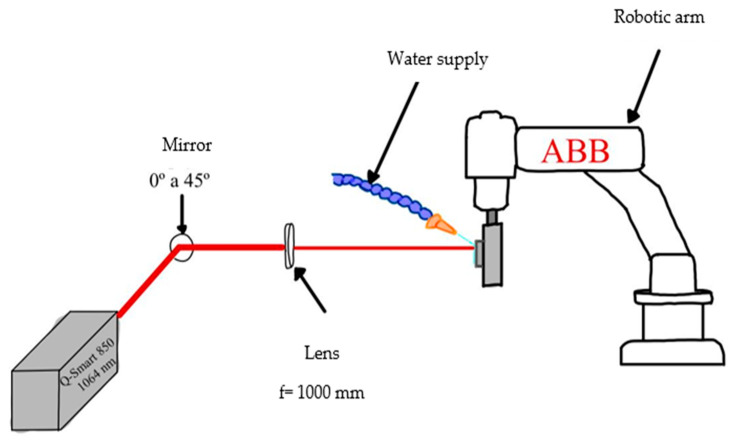
Experimental LSP setup.

The experimental setup is shown in Figure 2. The Quantel Q-smart 850 laser source (formerly Quantel, Les Ulis, France) is a class IV solid-state (Nd:YAG) laser, operating at a wavelength of 1064 nm, 6 nanoseconds pulse width, a repetition rate of 10 Hz with 0.750 J of energy per pulse with a beam diameter of 0.008 m (these parameters are based on the study by Reynoso-Garcia et al. [17]), with an almost Gaussian beam (Table 4) and a model ABB IRB-120 robotic arm (ABB Robotics, Västerås, Sweden). The laser pulse is redirected by an optical system consisting of a mirror and a THORLABS LA1464-YAG plano-convex lens (Thorlabs Inc., Newton, NJ, USA), manufactured from N-KB7 glass (Schott AG, Mainz, Germany).

**Figure 3:**
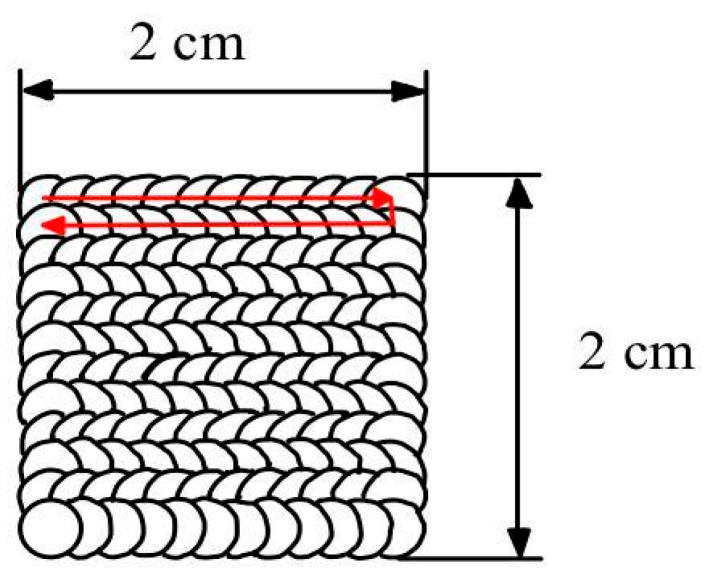
Area of application and direction of LSP treatment sweep. The arrows indicate the laser scanning path and the sequence of the overlapping spots.

The LSP treatment area for the WR Ti-6Al-4V and AM Ti-6Al-4V samples is 20 mm × 20 mm for both treatments, with a scanning density of 2500 pulses/cm^2^. The scanning directions and treatments are shown in Figure 3.

### 2.3. Characterization Methods

The test was subjected to X-ray fluorescence (XRF) testing to identify the chemical elements present. NEX QC + QUANTEZ RIGAKU (Rigaku Corporation, Akishima, Tokyo, Japan) XRF equipment was used with voltage settings ranging from 1 to 50 kV. X-ray diffraction (XRD) testing was used to identify the crystalline structure of the sample and the phases present in the titanium alloys. The equipment used was PARALYTICAL EMPYREAN (Malvern Panalytical (PANalytical), Almelo, The Netherlands) with a step size of 0.0001°, a θ_min of 20° and a θ_max of 80°, a wavelength of 1.54 angstroms with a Cu source, and a run time of 4:50 min.

To obtain results for residual stresses (RS) induced in the test piece by the treatment, the hole-drilling technique was used, with the Vishay RS-200 equipment (Milling Guide, Wendell, NC, USA). This test was performed in accordance with standard ASTM E-837-01 [31]. The RS test was performed under the following conditions: guide for R-200 model cutter, which will allow control of the precision of the cutter’s penetration depth into the sample using a micrometer (Mitutoyo, Kawasaki, Japan) incorporated into this equipment; to measure microvolt changes, the P3 micro deformation measuring device (Micro-Measurements, Raleigh, NC, USA) and a CEA-06-062UL-120 extensometer gauge (Micro-Measurements, Raleigh, NC, USA) were used. The quantitative error margin for the residual stress measurements was estimated to be within ± 5%. This measurement uncertainty is primarily influenced by the instrument precision of the RS-200 milling guide depth control (±0.025 mm) and the inherent sensitivity tolerance of the CEA-06-062UL-120 strain gauges.

Microhardness characterization was performed in accordance with ASTM E384-99 [32]. For areas with and without surface treatment in the cross section of the test piece, the parameters used for micro indentations were a load application time of 15 s and a test load of 0.98 N. The equipment used was the FM 800 microhardness tester (Future-Tech Corp, Kawasaki, Kanagawa, Japan). Microhardness measurements were performed according to the ASTM E384-99 standard across the cross-section of both the as-received and LSP-treated samples. To accurately determine the mechanical hardening gradient, indentation profiles were executed from the treated surface to a depth of 1 mm into the bulk material. The measurement points were precisely located within the central zone of the LSP interaction area; this ensures the capture of the material’s response under maximum energy flux density and plasma overlap, thereby guaranteeing the representativeness of the induced plastic deformation. Furthermore, an inter-indentation spacing greater than *3d* was strictly maintained to mitigate interference from adjacent strain hardening and residual stress fields. This systematic protocol allows for a direct correlation between the shockwave attenuation kinetics and the inherent microstructural disparities of each manufacturing process.

In order to characterize the changes in the microstructure of both parts, grain size tests were prepared based on the ASTM E3-11 standard [33], in which mechanical roughing was carried out. To reveal the microstructure, Kroll acid preparation was used. This solution was selected based on ASTM E407-23 standard [34], which is composed of 3% nitric acid (HNO3), 1% hydrofluoric acid (HF) and 96% distilled water. The acid was deposited on the surface for 20 s at a temperature of 20 °C.

The metallographic images were taken using a Nikon Eclipse optical microscope (Nikon, Tokyo, Japan) at 50× magnification; the phase fraction quantification was performed using automatic thresholding based on Otsu’s method, which determines variance between segmented regions [35]. The quantitative error associated with the phase fraction analysis via the Otsu thresholding method is estimated at ±3%. This error margin accounts for potential variations in local contrast, lighting conditions during image acquisition, and microstructural etching uniformity across the analyzed optical micrographs.

## 3. Results

### 3.1. XRF Results

The results of the X-ray fluorescence (XRF) characterization for the WR Ti-6Al-4V and AM Ti-6Al-4V samples are shown in Table 5.

Based on the results obtained by this characterization technique, we can identify that both test pieces have three main chemical elements: titanium, aluminum, and vanadium, with percentages that are characteristic of a Ti-6Al-4V alloy. These elements are within the typical ranges established by ASTM F1472 (Al: 5.50–6.75% and V: 3.50–4.50%) [25,36]. However, we can distinguish a clear difference in solute concentration between the two manufacturing processes.

The AM Ti-6Al-4V sample has a significantly higher aluminum concentration (6.75 wt%) than the WR sample (6.26 wt%), placing it at the upper limit permitted by the regulations. As this is an α-phase stabilizer, we can expect greater hardness compared to the WR Ti-6Al-4V sample if we assume similar cooling rates. For their part, the β-phase stabilizers in the Ti-6Al-4V AM sample show an enrichment in both vanadium (4.50 wt%) and iron (0.30 wt%) compared to the WR Ti-6Al-4V sample. This could imply a microstructure with slightly more stable retained β phases due to the higher V and Fe content.

### 3.2. XRD Results

**Figure 4:**
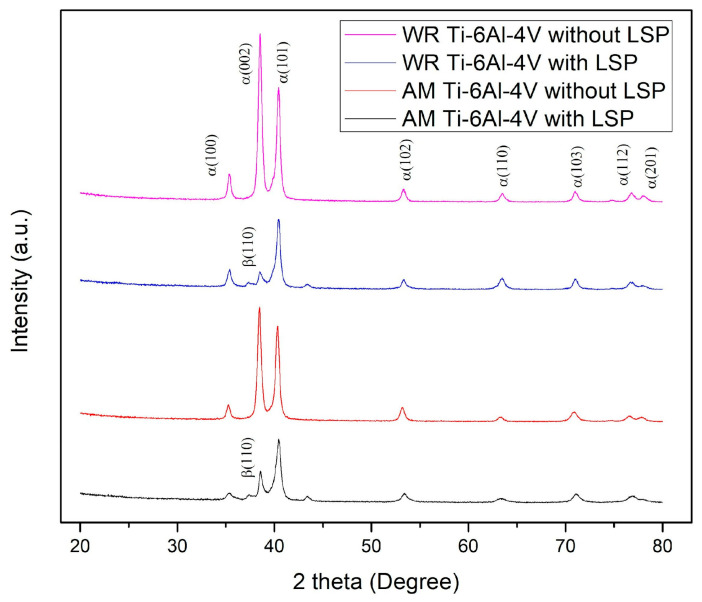
Diffractograms of AM Ti-6Al-4V and WR Ti-6Al-4V.

To confirm the identity of the material and the phases present in it, characterization was carried out using X-ray diffraction. The analysis showed the predominant presence of α-Ti (HCP) and β-Ti (BCC) phases in both pieces, which unequivocally indicates that we are dealing with a titanium-based alloy. The results are shown in Figure 4.

### 3.3. Residual Stress Results

**Figure 5:**
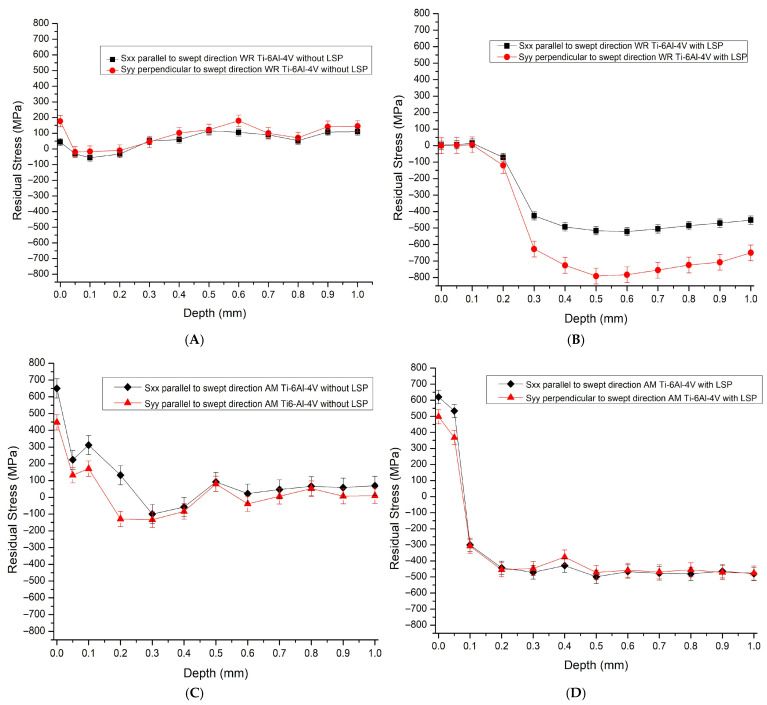
Residual stress curves, (**A**) WR Ti-6Al-4V without LSP, (**B**) WR Ti-6Al-4V with LSP, (**C**) AM Ti-6Al-4V without LSP and (**D**) AM Ti-6Al-4V with LSP.

Figure 5 shows the results of the residual stress generated by the LSP treatment on the WR Ti-6Al-4V and AM Ti-6Al-4V samples.

The curves in Figure 5A show the results for the WR Ti-6Al-4V sample without LSP treatment. We can confirm the presence of tensile residual stresses with a maximum value close to 200 MPa. This phenomenon is attributable to thermal gradients and heterogeneous plastic deformation accumulated during the wrought manufacturing process.

After applying the laser pulse impact treatment, the residual stress results change as follows (with black square markers for the direction parallel to the scan and red circular markers for the direction perpendicular to the laser treatment scan); the generation of compressive residual stresses is observed with a maximum field close to −800 MPa at a depth of 500 μm, indicating efficient propagation of the plastic wave through the laminar microstructure. In addition to this, values between 200 and 300 μm represent the interaction zone where permanent plastic deformation compensates for the intrinsic tensile stresses of the laminate.

Figure 5C shows the results obtained for the AM Ti-6Al-4V sample. We can see that the samples without LSP treatment show the typical profile of additive manufacturing. Extremely high tensile residual stresses are recorded on the surface, with a maximum field of +600 MPa. These stresses are the result of rapid heating and cooling cycles, which generate severe thermal gradients and restrict solid contraction during solidification.

Following the application of LSP treatment to the AM Ti-6Al-4V sample (Figure 5D), there is a reversal in the state of residual stress in the sample. At a depth of just 100 μm, the material undergoes a drastic transition from tension to compression; from 200 μm onwards, a plateau of compression residual stresses (CRS) of approximately −500 MPa is established; these curves show that the compression level remains almost constant until the end of the measured range (1000 μm). In addition to this, LSP treatment not only changes the sign of stress, but also acts as a mechanical homogenization process, reducing the intrinsic tensile anisotropy of the part’s creation.

### 3.4. Hardness

**Figure 6:**
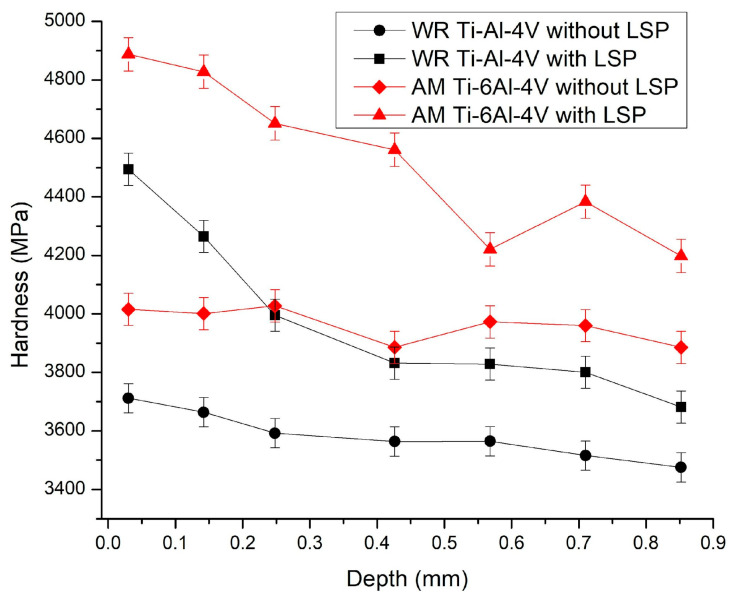
Hardness curve of Ti-6Al-4V samples.

The results of the hardness characterization are shown in Figure 6, where we can identify hardness curves representing the AM Ti-6Al-4V samples without LSP treatment (with red diamonds) and with LSP treatment (with red triangles); the curves for the WR Ti-6Al-4V parts without treatment (black circles) and with LSP treatment (black squares).

Microhardness measurements were performed at different depths from the treated surface. For each depth level, the mean value and standard deviation were calculated from the indentations performed. A total of 24 indentations were carried out per sample to ensure statistical reliability. The hardness curves for the AM Ti-6Al-4V test piece show a significant increase in hardness after undergoing LSP treatment, with maximum values of 4887.6 MPa and minimum values of 3924.4 MPa compared to the untreated sample, which shows values of ~4015.8 MPa at its maximum and 3885.4 MPa at its minimum. In both cases, microhardness values tend to decrease as depth increases, which is a characteristic behavior of samples that have undergone surface treatment.

The curves corresponding to the WR Ti6-AI-4V samples show values between 3711.8 MPa and 3475.5 MPa at their maximum and minimum points, respectively, which are typical values for the hardness of WR Ti-6Al-4V alloy parts that have not undergone any type of treatment. However, after undergoing LSP surface treatment, the microhardness values of the test pieces increase to 4494.4 MPa and 3681.4 MPa at their maximum and minimum points, respectively. It was observed that they tend to behave in the same way as the AM Ti-6Al-4V samples, decreasing as the depth increases.

### 3.5. Microstructure

**Figure 7:**
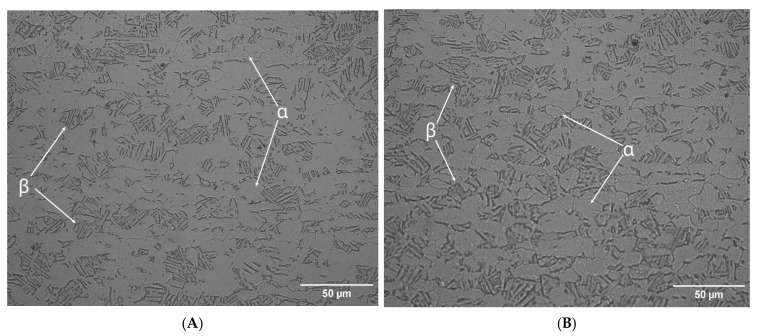
Otsu method image: (**A**) WR Ti-6Al-4V without LSP treatment and (**B**) WR Ti-6Al-4V with LSP treatment.

**Figure 8:**
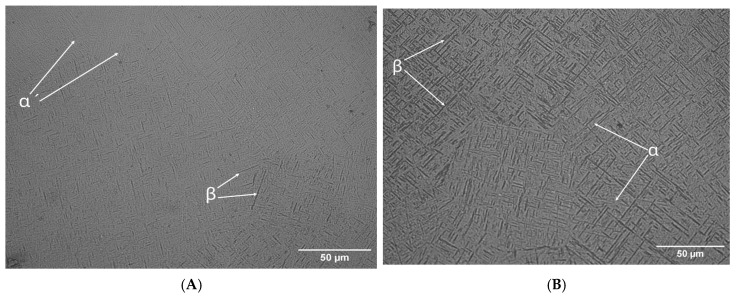
Otsu method image: (**A**) AM Ti-6Al-4V without LSP treatment and (**B**) AM Ti-6Al-4V with LSP treatment.

**Figure 9:**
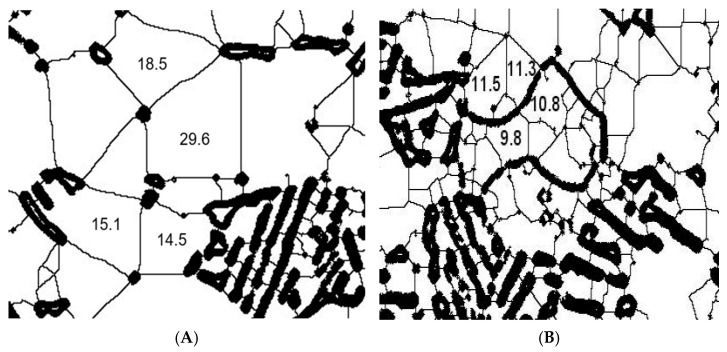
Grain size: (**A**) WR Ti-6Al-4V without LSP treatment and (**B**) WR Ti-6Al-4V with LSP treatment.

**Figure 10:**
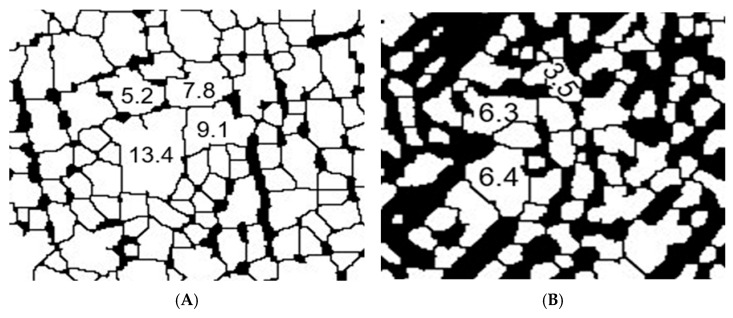
Grain size: (**A**) AM Ti-6Al-4V without LSP treatment and (**B**) AM Ti-6Al-4V with LSP treatment.

The effects of LSP treatment on the morphology of the Ti-6Al-4V alloy in samples of AM Ti-6Al-4V and WR Ti-6Al-4V are shown in Figure 7, Figure 8, Figure 9 and Figure 10. The evaluation focused on determining grain size using the planimetric method in accordance with ASTM E112 [37], as well as quantifying the percentage of α and β phases using digital image analysis in accordance with ASTM E1245 [38].

A quantitative phase analysis was performed using automatic thresholding (Otsu method) on Figure 6 in accordance with ASTM E1245 to determine the phase content in metallographic images of WR Ti-6Al-4V before and after LSP treatment. The result was an α phase percentage of 75.17% and a β phase percentage of 24.83% before surface treatment; these percentages are consistent with values reported for a Ti-6Al-4V sample. After LSP treatment, the phase percentages were reconfigured, yielding the following results: 72.23% α phase and 27.77% β phase, indicating an increase in the β phase after LSP treatment. 

Figure 8 shows the effects of LSP treatment on the AM Ti-6Al-4V sample. When subjected to Otsu analysis, we find that the α phase present in the sample is 81.5% and the β phase is 18.5% before the application of the treatment. The phase percentages after being subjected to LSP treatment are 45.3% and 54.7%, respectively, for each phase.

The grain size results before and after LSP treatment are shown in Figure 9. Figure 9A represents a section of the WR Ti-6Al-4V test piece without LSP treatment, with grain sizes ranging from 14.5 to 29.6 μm. 

Figure 10B shows a section of the AM Ti-6Al-4V test piece with LSP treatment, where grain sizes ranging from 9.9 to 11.3 μm were obtained. For WR Ti-6Al-4V without LSP treatment, the grain size ranges from 5.10 to 20. Once the LSP treatment is applied, the grain size changes to between 2.5 and 5.60 μm. (The images were processed using IMAGEJ software: version 1.54g, National Institutes of Health, Bethesda, MD, USA).

However, to confirm the findings shown in Figure 9 and Figure 10, a statistical analysis of the size of each grain was performed. There is a change in morphology in the microstructure of both pieces, while the WR Ti-6Al-4V piece shows a granular morphology in the alpha phase. The AM Ti-6Al-4V piece shows a lamellar (Widmanstätten) morphology that, in both cases, is refined after being subjected to the LSP treatment. The grain size results for AM Ti-6Al-4V and WR Ti-6Al-4V samples with and without LSP are shown in Table 6.

Based on the results shown in Table 6, we can infer grain refinement once the LSP treatment is applied, since an increase in the ASTM G column implies a refinement in the grain size of the sample, which generally improves dynamic mechanical properties such as fatigue or toughness.

## 4. Discussion

The results obtained in the X-ray diffraction tests for both samples analyzed show an excellent correlation between the position and intensity of the diffraction peaks, indicating that both pieces have the same crystalline structure. In both diffractograms, we can see characteristic peaks at angles of 2θ in positions close to 35.1°, 38.5°, 40.0°, 53.2°, 63.1°, and 70.9°. The presence and precise position of these peaks in both samples suggest that, despite being different pieces, they are made of the same base material with an equivalent crystalline structure [39]. This coincides with the standard pattern reported in the ICDD PDF-2 database for Ti-6Al-4V material.

For their part, the residual stress results obtained using the hole-drilling technique show that compressive residual stresses of around −500 MPa are generated for the AM Ti-6Al-4V piece at a depth of 1.0 mm. These values may be related to the porosity present in the material, an effect based on the findings published by Dyer et al. [19], who present behavior similar to that reported in this study, but with values of −400 MPa that are slightly lower than those obtained, although they coincide with the response of the LSP treatment applied to porous materials. However, the samples exhibit a critical surface tensile stress of approximately 650 MPa, which is characteristic of the extreme thermal gradients inherent to the SLM process. Following the application of coating-less LSP, the results demonstrate that the direct thermal interaction of the laser pulse with the inherent roughness of the additive manufacturing surface maintains this tensile state within the first 200 µm. This phenomenon is driven by the micro-melting and subsequent contraction of surface asperities, a mechanism extensively discussed by Dyer et al. [19], regarding the interaction between high-energy laser pulses and the complex topography of AM alloys.

As for the WR Ti-6Al-4V piece, residual compressive stress values were obtained with maximum values of −800 MPa at a depth of 0.6 mm. These results indicate that the LSP treatment was successfully applied to the sample. This assertion is based on the findings reported by Ouyang et al. [40]. Their work indicates that LSP treatment applied to WR Ti-6Al-4V induces compressive residual stresses of up to −800 MPa penetrating approximately 0.62 mm depth. In both studies, there is high anisotropy between the residual stress curves. In this context, it is concluded that although both studies achieve comparable levels of residual compression, Ouyang’s model shows a more homogeneous and predictable stress field, while the experimental sample presents a more marked anisotropy, representative of real industrial application conditions.

The Vickers hardness (HV) results show a positive effect on both parts. The AM Ti-6Al-4V sample with LSP shows the greatest increase in hardness (~4903.3 MPa) compared to the AM Ti-6Al-4V without LSP (~4020.7 MPa), representing an approximate increase of 22%. This can be attributed to the introduction of residual compressive stresses and a possible microstructural refinement that would increase resistance to plastic deformation. That this phenomenon was observed after treatment may be associated with microstructural refinement and increased crystalline defect density, consistent with reports by Ma, X. et al. [41] on Ti-6Al-4V subjected to severe deformation, where the increase in microhardness is directly linked to grain subdivision and dislocation accumulation. For WR Ti-6Al-4V samples with LSP, there is also an increase in hardness (~4511.1 MPa) after undergoing LSP treatment (WR Ti-6Al-4V without LSP: ~3677.5 MPa), indicating a 23% increase, showing that the starting values are lower than in the AM Ti-6Al-4V without LSP. The Vickers microhardness results reveal an exceptional mechanical response of Ti-6Al-4V to LSP treatment, exceeding the thresholds reported in conventional literature; an example of this is the reports by W. Okuniewski et al. [42]. In their research, they establish a base hardness for wrought material of ~3334.3 MPa. In comparison, our WR Ti-6Al-4V samples with LSP achieve a 15% increase typically observed in equiaxed structures α+β; the results for AM Ti-6Al-4V achieved a value of 4903.3 MPa after post-processing, representing a 22% increase over the as-built state of 4020.7 MPa. This behavior exceeds the findings of A. Khorasani et al. [43], who attributed the superior hardness of AM material to the acicular martensitic phase α′, but who place the increase due to LSP in lower ranges. The results obtained in this research suggest extreme microstructural refinement and a density of dislocations induced by the shock wave. Based on what has been reported by. L. Lian et al. [44], this indicates that it is enhanced by the intrinsic fine columnar morphology of additive manufacturing; achieving a resistance to plastic deformation greater than that of the wrought material.

This difference may be related to the initial microstructure: AM Ti-6Al-4V typically has a finer, more columnar structure that is more susceptible to modification by LSP treatment than the more equiaxial and stable microstructure of WR Ti-6Al-4V.

This is closely related to the grain size of Ti-6Al-4V under different conditions, revealing a clear influence of both the manufacturing method and LSP treatment on the microstructure of the material. The WR Ti-6Al-4V sample without LSP has a larger than average grain size of 26.51 μm corresponding to an ASTM grain number of 9.90, while the Ti-6Al-4V AM sample without LSP shows a very fine grain size of 18.16 μm with an ASTM number of 10.45; this can be explained by the high thermal gradients and rapid solidification rates inherent in the AM process, which favors dense nucleation and limited grain growth. After undergoing LSP treatment, both samples show grain refinement. For the WR Ti-6Al-4V sample with LSP, the size was reduced from 26.51 to 18.16 µm (increase in ASTM number from 9.90 to 10.81) which represents a decrease of 31.5%. This trend is higher than that reported by Ren et al. [45], who recorded a reduction of 27.7% (from 33.6 to 24.3 µm). The higher refinement rate observed in this study suggests a denser activation of multidirectional mechanical twins and a subdivision of the phase. The grain refinement reported in our research can be attributed to the mechanical action of the shock waves generated by the LSP, which cause severe plastic deformation on the surface, promoting the formation of new sub-structures and possible processes of localized recrystallization or sub-granulation. For its part, the AM Ti-6Al-4V sample with LSP shows a constant grain size of 5.54 μm and an ASTM number change of 12.17. This indicates that this piece has a metastable α′ (martensite) structure due to the high cooling and solidification rates generated in the laser-based directed energy deposition (LB-DED) additive manufacturing technique, which promotes the conversion of the β phase of Ti-6Al-4V AM into the aforementioned structure [21,44]; this phenomenon results in acicular martensite within β columnar grains, as shown in Figure 7. LSP treatment induces severe plastic deformation on the surface, promoting fragmentation and refinement of α′ needles, as well as the formation of sub grains. One of the factors to consider is the increase in temperature due to the adiabatic conditions of high deformation speed, which is a key factor in material instability and a common precursor to local microstructural transformations [46]. The results suggest that LSP treatment could be associated with a redistribution of the volumetric fractions of the phases in the Ti-6Al-4V alloy, with a tendency towards an increase in the phase β. The results obtained by Otsu analysis show that AM Ti-6Al-4V parts have greater growth of this phase, this effect could be attributed to the interaction between the laser-induced shock waves and the initial microstructure of the AM-processed material, which appears to favor phase reconfiguration. However, the magnitude of this transition highlights the sensitivity of the alloy to surface post-processing, raising the possibility that LSP acts as a microstructural modification mechanism beyond the mere induction of residual stresses.

## 5. Conclusions

From this research, in which we compared the effects of LSP treatment when applied to traditionally manufactured and additively manufactured parts, we can draw the following conclusions:

The chemical and structural identity of both samples was validated through XRD and XRF analysis. The characteristic diffraction peaks and elementals percentages (Al 6%, V 4%, Ti bal.) showed a total correlation with the nominal Ti-6Al-4V alloy specifications. This confirms that the observed variations in post-LSP mechanical behavior are strictly attributable to the manufacturing process and its resulting microstructure, rather than chemical disparities.

LSP treatment successfully induced compressive residual stresses (CRS) in both manufacturing conditions, albeit with distinct profiles:

- WR Ti-6Al-4V samples achieved a maximum CRS magnitude of −750 MPa. The profile exhibited high localization and significant anisotropy between the principal directions, characteristic of the texture in forged materials.
- AM Ti-6Al-4V samples displayed a more isotropic and uniform response, but with a lower maximum magnitude of −450 MPa. This attenuation may be attributed to the scattering of the plasma-induced shock wave by the inherent porosity and complex microstructural boundaries of the additive manufacturing process.

The LSP-induced severe plastic deformation led to a significant hardening effect governed by the Hall–Petch relation:

- WR Ti-6Al-4V samples increased microhardness from 3677.5 MPa to 4511.1 MPa (22.6%) through a Hall–Petch grain refinement from 26.51 μm to 14.09 μm (ASTM G 9.09 to 10.82). The dense, equiaxial wrought microstructure facilitated efficient shock wave propagation with minimal attenuation, inducing severe plastic deformation (SPD) and high dislocation density. This resulted in a sustained hardening gradient at depths of 0.7–0.8 mm which, coupled with a peak compressive residual stress of −750 MPa, substantially improves the surface integrity and fatigue resistance of the material.
- AM Ti-6Al-4V samples induced a substantial hardening effect, increasing microhardness from 4020.7 MPa to 4903.3 MPa. This strengthening is directly correlated with a significant Hall–Petch grain refinement, reducing the mean grain size from 18.16 μm to 5.54 μm (ASTM G 10.45 to 12.17). Despite the inherent microstructural boundaries and porosity of the additive process, which scattered the shock wave and limited the peak compressive residual stress to −450 MPa, the high strain rate energy successfully triggered a severe plastic deformation regime. This resulted in a sustained hardening gradient at subsurface depths of 0.7–0.8 mm, confirming the effectiveness of LSP in enhancing the mechanical integrity of additively manufactured components.

LSP triggered a microstructural reconfiguration, specifically increasing the β-phase fraction. While the WR samples showed a moderate increment, the AM samples exhibited a substantial phase shift. This suggests that the high strain rate energy of the LSP shock wave interacts with the metastable microstructure of the AM alloy (e.g., martensitic α′) promoting an α′ -> β phase transition as quantified by Otsu thresholding analysis.

Future work will expand upon these findings by conducting a multiscale microstructural analysis. EBSD will be employed to quantify phase distribution, and TEM will provide insight into dislocation density and deformation mechanisms induced by the laser shock wave. Furthermore, the impact of LSP treatment on the functional performance of Ti-6Al-4V alloy will be evaluated through fatigue life testing and corrosion resistance assays to validate its suitability for industrial applications.

## Figures and Tables

**Table 1:** Chemical composition of WRTi-6Al-4V [21].

Element	Al	V	Fe	O	C	N	Y	Ti
wt%	6.75	4.50	0.30	0.20	0.08	0.05	0.005	Bal.

**Table 2:** Mechanical properties of WR Ti-6Al-4V [20].

Category	Chemical Composition	Hardness (HV)	Ys (MPa)	TS (MPa)	E (GPa)	$T_{\beta }\;\left({}^{\circ}C\right)$
α + β	Ti-6Al-4V	300–400	800–1100	900–1200	110–140	995

$T_{\beta }\;\left({}^{\circ}C\right)$ = *Phase change temperature*.

**Table 3:** Ti-6Al-4V alloy powder chemical composition.

Study Sample	Ti	Al	V	Fe	O	C	N	H
ASTM F3001-14	Bal.	5.5–6.5	3.5–4.5	0.25	0.13	0.08	0.05	0.0012
Ti-6Al-4V alloy powder	Bal.	6.17	3.48	-	0.11	-	0.0072	0.0012

**Table 4:** Operating parameters of the Q-smart 850 laser source.

Parameters	Values
Energy pulse (J)	0.750
Wavelength (nm)	1064
FWHM (ns)	6
Spot radius (mm)	0.5
Power density (GW/cm^2^)	15.92
Material/confining layer	Ti-6Al-4V/Water

**Table 5:** Chemical composition of Ti-6Al-4V samples.

Element	Al	V	Fe	Ag	Cu	Ni	Mo	Ti
AM Ti-6Al-4V (wt%)	6.75	4.50	0.30	0.002	0.034	0.070	0.010	88.11
WR Ti-6Al-4V (wt%)	6.26	4.20	0.230	0.002	0.019	0.063	0.002	89.20

**Table 6:** Grain size of Ti-6Al-4V.

Sample	Grain Numbers	Size (μm)	ASTM G
WR Ti-6Al-4V without LSP	661	26.51	9.09
WR Ti-6Al-4V with LSP	644	14.09	10.82
AM Ti-6Al-4V without LSP	11,641	18.16	10.45
AM Ti-6Al-4V with LSP	11,883	5.54	12.17

## Data Availability

The original contributions presented in this study are included in the article. Further inquiries can be directed to the corresponding author.
